# Digital health interventions for cognitive function in breast cancer patients treated with chemotherapy: a network meta-analysis of subjective and objective cognitive outcomes

**DOI:** 10.3389/fonc.2026.1989710

**Published:** 2026-10-29

**Authors:** Ruihong Zeng, Xiangyu Liu, Yujia Fan, Sijie Wu, Yaxuan Hu

**Affiliations:** 1 School of Nursing, Hunan University of Chinese Medicine, Changsha, China; 2 Department of Hospital Infection Management, Hunan Cancer Hospital, Changsha, China

**Keywords:** breast cancer, chemotherapy, cognitive function, cognitive rehabilitation, digital health, network meta-analysis

## Abstract

**Purpose:**

To systematically compare the effects of different digital health interventions on subjective and objective cognitive function in breast cancer patients treated with chemotherapy and to provide evidence for tailored supportive care and rehabilitation strategies.

**Methods:**

Eight databases, including the Cochrane Library, PubMed, Web of Science, Embase, CNKI, WanFang, VIP, and SinoMed, were searched from inception to March 2026 for randomized controlled trials (RCTs) evaluating digital health interventions for cognitive function in breast cancer patients treated with chemotherapy. Two reviewers independently screened studies, extracted data, and assessed risk of bias. A frequentist network meta-analysis was conducted using RevMan 5.3 and Stata 16.0. Interventions were ranked according to the surface under the cumulative ranking curve (SUCRA). The certainty of evidence was evaluated using the GRADE approach.

**Results:**

Twenty-two RCTs involving 1, 494 participants were included. Network meta-analysis (NMA) showed that digital exercise intervention (DEI) (SMD = 2.53, 95% CI 1.58 to 3.48) and digital psychological and behavioral intervention (DPBI) (SMD = 0.44, 95% CI 0.03 to 0.85) significantly improved subjective cognitive function compared with the control group (CON). DEI was also significantly more effective than digital multimodal intervention (DMI), DPBI, digital rehabilitation intervention (DRI), and digital cognitive training (DCT), and ranked highest (SUCRA = 100.0%). For objective cognitive function, DRI significantly improved cognitive function compared with CON (SMD = 2.59, 95% CI 1.17 to 4.02) and ranked highest (SUCRA = 85.5%), whereas no significant differences were observed in the remaining comparisons. The certainty of evidence was low.

**Conclusion:**

Digital health interventions may improve cognitive function in breast cancer patients treated with chemotherapy, with DEI showing the greatest potential for subjective cognitive function and DRI for objective cognitive function. However, these findings should be interpreted cautiously because of the low certainty of evidence.

**Systematic review registration:**

https://www.crd.york.ac.uk/PROSPERO/, identifier CRD420261331780.

## Introduction

Breast cancer is the most frequently diagnosed malignancy among women worldwide. According to the latest statistics from the International Agency for Research on Cancer (IARC), approximately 2.4 million new cases are diagnosed annually, accounting for about 23.2% of all cancers in women (1). Advances in early detection and treatment have substantially improved survival outcomes (2). As a result, increasing attention has shifted from survival alone to the management of treatment-related symptoms and long-term functional outcomes. Among these, chemotherapy-related cognitive impairment (CRCI) has emerged as an important issue in supportive and survivorship care for patients with breast cancer.

CRCI typically manifests as memory decline, impaired concentration, executive dysfunction, and reduced information processing speed (3). Previous studies have reported that 16% to 75% of breast cancer patients receiving chemotherapy experience some degree of cognitive impairment (4), and cognitive symptoms may persist for years after treatment completion in a substantial proportion of patients (5). The mechanisms underlying CRCI are complex and likely multifactorial, involving neuroinflammation, oxidative stress, neuronal injury, and other treatment-related biological processes (6, 7). Existing evidence suggests that CRCI not only compromises patients’ quality of life but also aggravates emotional distress, impairs social functioning and work capacity, and ultimately complicates the overall rehabilitation process (8, 9). Therefore, preventing cognitive decline and improving cognitive function have become important priorities across the continuum of breast cancer care.

Conventional approaches aimed at improving cognitive function, including face-to-face cognitive training, cognitive behavioral therapy, and pharmacological treatment, have shown potential benefits (10);however, their implementation in routine care is often limited by barriers related to time, location, and healthcare resources (11). For breast cancer patients who have received chemotherapy, treatment-related symptoms such as fatigue, emotional distress, and reduced physical functioning may further restrict access to sustained cognitive support. In this context, digital health interventions have gained increasing interest as potentially accessible and scalable approaches for cognitive rehabilitation and supportive care (12). By reducing spatial and temporal barriers, digital interventions may improve the accessibility, flexibility, and continuity of cognitive support and rehabilitation for patients treated with chemotherapy (13).

Digital health interventions targeting cognitive function in breast cancer patients treated with chemotherapy encompass a range of approaches, including digital cognitive training, digital rehabilitation, digital psychological and behavioral interventions, digital exercise interventions, and digital multimodal interventions. These interventions may be tailored to individual symptom burden, functional status, and rehabilitation needs, thereby potentially improving engagement and feasibility in clinical practice. Previous studies have evaluated web-based cognitive training and remotely delivered exercise interventions for cognitive outcomes in breast cancer populations (14, 15). However, findings have varied across intervention types and cognitive outcome measures, and the relative effects of different digital intervention modalities remain unclear.

Nevertheless, the current evidence base remains fragmented. Most previous studies have focused on individual intervention types, and direct comparisons across different digital health interventions are scarce. As a result, it remains unclear whether different digital health interventions have differential effects on subjective and objective cognitive function and which intervention modality may provide greater benefit for breast cancer patients who have received chemotherapy. This distinction is clinically relevant because subjective and objective cognitive outcomes reflect related but distinct aspects of cognitive function, and intervention effects may differ between these outcome domains.

Therefore, we conducted a network meta-analysis (NMA) to compare the effects of different digital health interventions on subjective and objective cognitive function in breast cancer patients treated with chemotherapy. By integrating direct and indirect evidence, this study aimed to compare the relative effects of different intervention modalities and summarize their ranking probabilities, thereby providing evidence for cognitive supportive care and rehabilitation in this population.

## Methods

### Protocol and registration

This network meta-analysis was conducted in accordance with the Preferred Reporting Items for Systematic Reviews and Meta-Analyses for Network Meta-Analyses (PRISMA-NMA) statement and the methodological recommendations of the Cochrane Handbook for Systematic Reviews of Interventions. The protocol for this study was registered in the International Prospective Register of Systematic Reviews (PROSPERO) under registration number [CRD420261331780].

### Literature search strategy

A comprehensive and systematic literature search was conducted across both English and Chinese electronic databases, including PubMed, Web of Science, the Cochrane Library, Embase, China National Knowledge Infrastructure (CNKI), Wanfang Data, the VIP Database for Chinese Technical Periodicals (VIP), and the Chinese Biomedical Literature Service System (SinoMed). All databases were searched from their inception to March 2026. To ensure the completeness of the search, the reference lists of relevant systematic reviews and meta-analyses were manually screened. In addition, the reference lists of all included studies were checked to identify any additional eligible articles.

### Eligibility criteria

The eligibility criteria were established according to the PICOS framework (Population, Intervention, Comparator, Outcomes, and Study design). The inclusion criteria were as follows: (P) adults aged ≥18 years with clinically or pathologically confirmed breast cancer who were undergoing or had completed chemotherapy; (I) digital health interventions, including digital cognitive training, rehabilitation, psychological or behavioral interventions, exercise-based interventions, and multimodal interventions, delivered through computers, websites, mobile applications, telehealth systems, or other digital platforms; (C) comparator conditions, including usual care, standard care, health education, conventional cognitive intervention, wait-list control, no intervention, or sham interventions; (O) at least one subjective or objective measure of cognitive function, such as the Functional Assessment of Cancer Therapy–Cognitive Function (FACT-Cog), Patient-Reported Outcomes Measurement Information System (PROMIS) cognitive measures, Cognitive Failures Questionnaire (CFQ), cognitive functioning subscale of the EORTC QLQ-C30, Montreal Cognitive Assessment (MoCA), or other validated neuropsychological tests; and (S) randomized controlled trials (RCTs).

### Classification of interventions and network nodes

Interventions were classified into five digital health intervention nodes and one control node according to their primary therapeutic components, intervention objectives, and delivery characteristics. The control group (CON) included usual care, standard care, conventional cognitive intervention, wait-list control, health education, or sham interventions.

Digital cognitive training (DCT) included computerized, web-based, or application-based interventions primarily designed to improve cognitive domains, such as memory, attention, processing speed, and executive function, through structured cognitive exercises.

Digital rehabilitation intervention (DRI) referred to technology-supported rehabilitation programs delivered through digital platforms, telehealth systems, or other digital approaches, aiming to improve cognitive function, functional performance, or participation in daily activities.

Digital psychological and behavioral intervention (DPBI) included digitally delivered psychological or behavioral approaches focusing on emotional regulation, stress management, coping strategies, and behavioral modification.

Digital exercise intervention (DEI) included digitally delivered physical activity or exercise-based programs designed to promote physical function, exercise participation, and cognitive-related outcomes through remote guidance or digital platforms.

Digital multimodal intervention (DMI) comprised digital interventions integrating two or more components, such as cognitive training, physical exercise, psychological support, health education, or self-management strategies.

Each intervention category was predefined as an independent node in the network meta-analysis.

### Literature selection and data extraction

All retrieved records were imported into NoteExpress software for duplicate removal. After deduplication, two reviewers independently screened titles and abstracts according to the predefined eligibility criteria. The full texts of potentially relevant studies were then assessed to determine final inclusion.

Data extraction was independently performed by the same two reviewers using a standardized form. The extracted data included: (1) study characteristics, including first author, publication year, and country; (2) participant characteristics, including sample size and age; (3) intervention characteristics, including intervention type, duration, and comparator; (4) outcome data, including assessment tools and cognitive outcome scores.

Any disagreements during study selection or data extraction were resolved through discussion. If consensus could not be reached, a third reviewer was consulted.

### Risk of bias assessment

The methodological quality of the included studies was assessed independently by two reviewers using the Cochrane Risk of Bias tool. The following domains were evaluated: random sequence generation, allocation concealment, blinding of participants and personnel, blinding of outcome assessment, incomplete outcome data, selective reporting, and other sources of bias.

Any discrepancies in the assessment were resolved through discussion or consultation with a third reviewer.

### Statistical analysis

Conventional pairwise meta-analyses were performed using Review Manager (RevMan), version 5.3. Statistical heterogeneity across studies was assessed using the I² statistic. Given the anticipated clinical and methodological heterogeneity among the included studies, random-effects models were applied. As cognitive outcomes were continuous and measured using different instruments, standardized mean differences (SMDs) with 95% confidence intervals (CIs) were calculated as the summary effect estimates. Before pooling, the direction of outcome scores was harmonized so that higher scores consistently represented better cognitive function; accordingly, positive SMDs indicated effects favoring the intervention group.

A frequentist network meta-analysis was conducted using Stata version 16.0, with random-effects models used as the primary analytical approach. Network geometry plots were constructed to illustrate the available direct comparisons. In the network plots, the size of each node was proportional to the total sample size receiving the corresponding intervention, whereas the thickness of the connecting lines reflected the number of studies contributing to each direct comparison.

For treatment networks containing closed loops, inconsistency between direct and indirect evidence was assessed, with P > 0.05 indicating no statistically significant inconsistency. The relative efficacy of the different interventions was ranked using the surface under the cumulative ranking curve (SUCRA), with higher SUCRA values indicating a greater probability of an intervention being among the top-ranked treatments.

Comparison-adjusted funnel plots were constructed to preliminarily assess potential publication bias and small-study effects. Sensitivity analyses were performed to evaluate the robustness of the network meta-analysis findings. First, network-specific leave-one-out analyses were conducted by sequentially excluding each individual study to assess the influence of single studies on effect estimates, treatment rankings, and between-study heterogeneity. Second, fixed-effect models were applied as an additional sensitivity analysis to examine whether the main conclusions were sensitive to the choice of statistical model. Given the anticipated clinical and methodological heterogeneity among the included studies, random-effects models were retained as the primary analyses.

### Certainty of evidence

The certainty of evidence was evaluated by two independent reviewers utilizing the Grading of Recommendations Assessment, Development and Evaluation (GRADE) approach. The certainty of evidence was classified into one of four categories: high, moderate, low, or very low. In accordance with the GRADE framework, evidence derived from randomized controlled trials (RCTs) was initially rated as “high” certainty. This rating was subsequently subject to downgrading based on five pre-specified domains: risk of bias, inconsistency, indirectness, imprecision, and publication bias.

## Results

### Literature search results

A total of 1, 757 records were initially identified through the database search. After removing 681 duplicate records, 1, 076 citations remained for title and abstract screening, and 1, 011 records were excluded based on the predefined eligibility criteria. The full texts of the remaining 65 articles were assessed for eligibility, resulting in the exclusion of 43 articles due to unavailable full texts, non-breast cancer populations, incomplete data, inappropriate interventions or outcomes, and duplicated sample sources. Finally, 22 randomized controlled trials met the eligibility criteria and were included in the network meta-analysis. The study selection process is presented in **Figure 1**.

**Figure 1:**
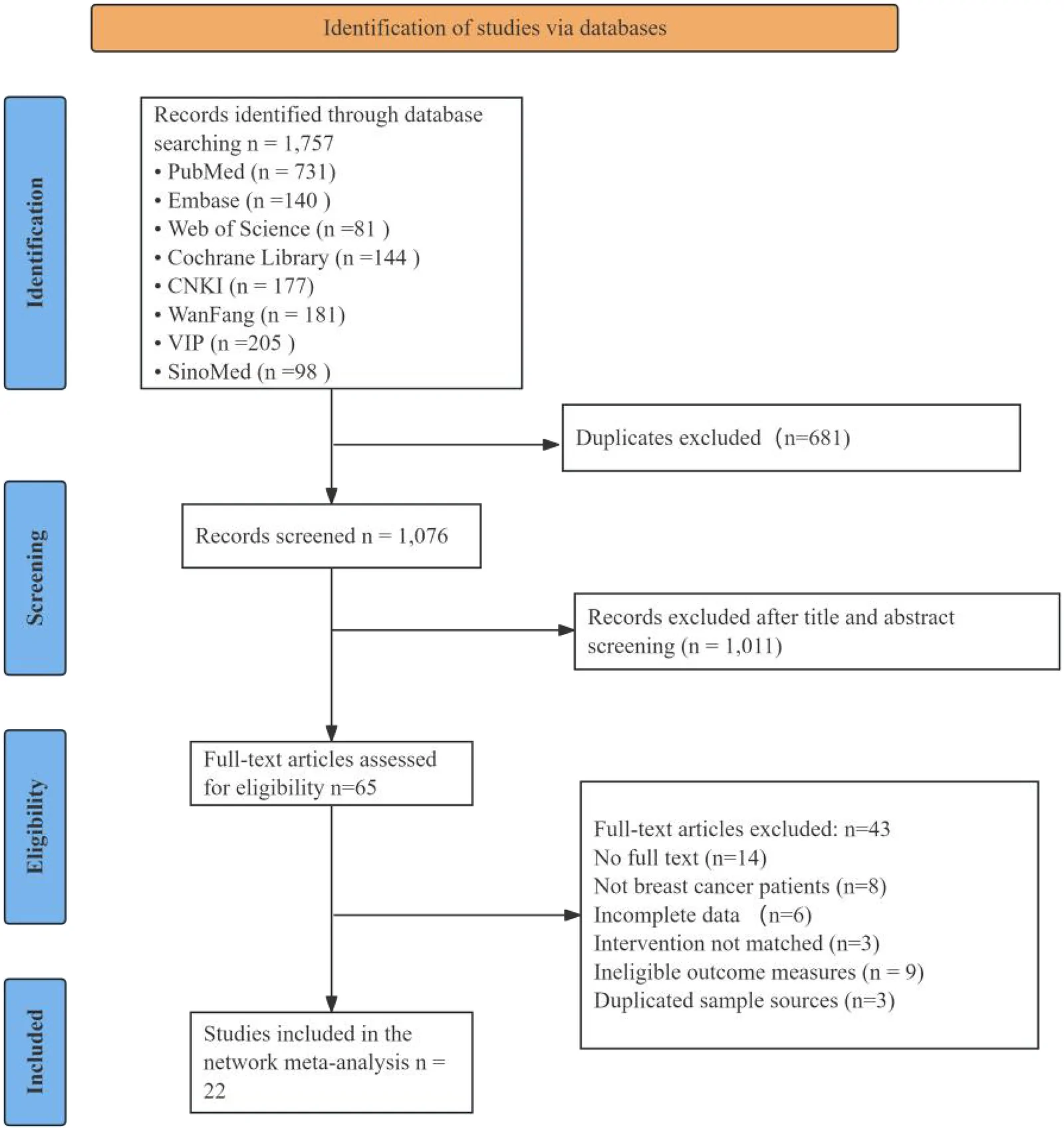
Flowchart of the systematic literature review.

### Characteristics of included studies

A total of 22 RCTs (14, 16–36), involving 1, 494 participants, were included. The included studies evaluated five digital health intervention categories, including digital cognitive training (DCT), digital rehabilitation intervention (DRI), digital psychological and behavioral intervention (DPBI), digital exercise intervention (DEI), and digital multimodal intervention (DMI), with control group (CON) as the reference node. Each intervention category was treated as an independent node in the network meta-analysis. The baseline characteristics of the included studies are summarized in **Table 1**.

**Table 1:** Basic characteristics of the included studies.

First author & Year	Study country	Age (T/C)	Sample size		Interventions		Duration	Outcomes
			T	C	T	C		
Guo 2023	China	48.96 ± 9.82/51.92 ± 8.49	26	26	DEI	CON	12 weeks	1, 2
Wang 2023	China	48.46 ± 6.93/47.77 ± 6.45	61	61	DMI	CON	5 months	2
Liu 2022	China	50.64 ± 7.28/49.82 ± 7.51	50	51	DMI	CON	4 months	2
Gao 2018	China	52.08 ± 10.0	40	40	DRI	CON	4 months	2
Yang 2023	China	59 ± 6.5/50.93 ± 5.89	52	52	DRI	CON	8 weeks	2
Wang 2024	China	49.71 ± 6.10/49.48 ± 5.61	28	29	DPBI	CON	6 weeks	1
Jin 2020	China	47.29 ± 11.41/47.83 ± 8.18	24	23	DRI	CON	8 weeks	2
Yang 2021	China	52.4 ± 4.1/52.8 ± 4.4	48	48	DRI	CON	8 weeks	2
Chapman 2023	UK	34~60/36~61	24	24	DCT	CON	2 weeks	1
Kleinknecht 2024	Germany	53.6 ± 11.2/54.4 ± 13.8	16	15	DCT	CON	14 weeks	1
Loubani 2021	Canada	48.00 ± 11.14/52.06 ± 12.8	18	17	DRI	CON	6 weeks	1, 2
Von Ah 2022	USA	56.3 ± 9.3/58.8 ± 6.7	19	17	DCT	CON	10 weeks	1
Bahçaci 2024	Turkey	49.1 ± 8.8/44.7 ± 10.0	26	26	DPBI	CON	6 weeks	1
Song 2018	China	52.1 ± 10.1/51.1 ± 10.1	40	40	DRI	CON	4 months	2
Wang 2021	China	50.86 ± 3.24/50.89 ± 3.21	28	29	DMI	CON	12 weeks	2
Damholdt 2016	Denmark	54.98 ± 8.51/54.56 ± 8.74	94	63	DCT	CON	6 weeks	1
Freeman 2015	USA	55.44 ± 8.08/55.57 ± 9.88	23	48	DPBI	CON	5 weeks	1
Sun 2021	China	53.05 ± 5.02/51.53 ± 4.45	55	55	DMI	CON	4 weeks	1
Ferguson 2016	USA	54.0 ± 12.82/55.61 ± 11.39	22	13	DPBI	CON	8 weeks	1
Smith 2021	USA	31~72	16	9	DCT	CON	4 weeks	1
Bellens 2020	Belgium	51.5 ± 8.0/52.1 ± 9.1	23	23	DCT	CON	12 weeks	1, 2
Myers 2020	USA	56.96 ± 11.49/54.31 ± 10.07	26	26	DRI	CON	6 weeks	1

T, treatment group; C, control group. Outcomes: 1, subjective cognitive function; 2, objective cognitive function. Interventions: CON, control group; DCT, digital cognitive training; DRI, digital rehabilitation intervention; DMI, digital multimodal intervention; DPBI, digital psychological and behavioral intervention; DEI, digital exercise intervention.

### Risk of bias assessment

The methodological quality of the included studies was assessed using the Cochrane Risk of Bias tool. Specifically, 16 trials (14, 16–21, 24, 26, 28–34) explicitly reported adequate methods for random sequence generation, 4 trials (21, 26, 33, 34) clearly described allocation concealment, and 6 trials (14, 17, 26, 29, 34, 36) implemented blinding of outcome assessment; these were consequently rated as having a low risk of bias in their respective domains. Studies lacking sufficient details regarding their randomization or blinding procedures were classified as having an unclear risk of bias.

Notably, owing to the inherent nature of the digital health interventions, blinding of participants and personnel was largely unfeasible across the majority of the trials, resulting in a predominantly high risk of performance bias. Additionally, two studies (21, 25) were rated as high risk for incomplete outcome data due to substantial attrition rates, whereas the remaining trials were judged as low risk in this regard.The detailed risk of bias summary and graph are presented in **Figures 2**, **3**.

**Figure 2:**
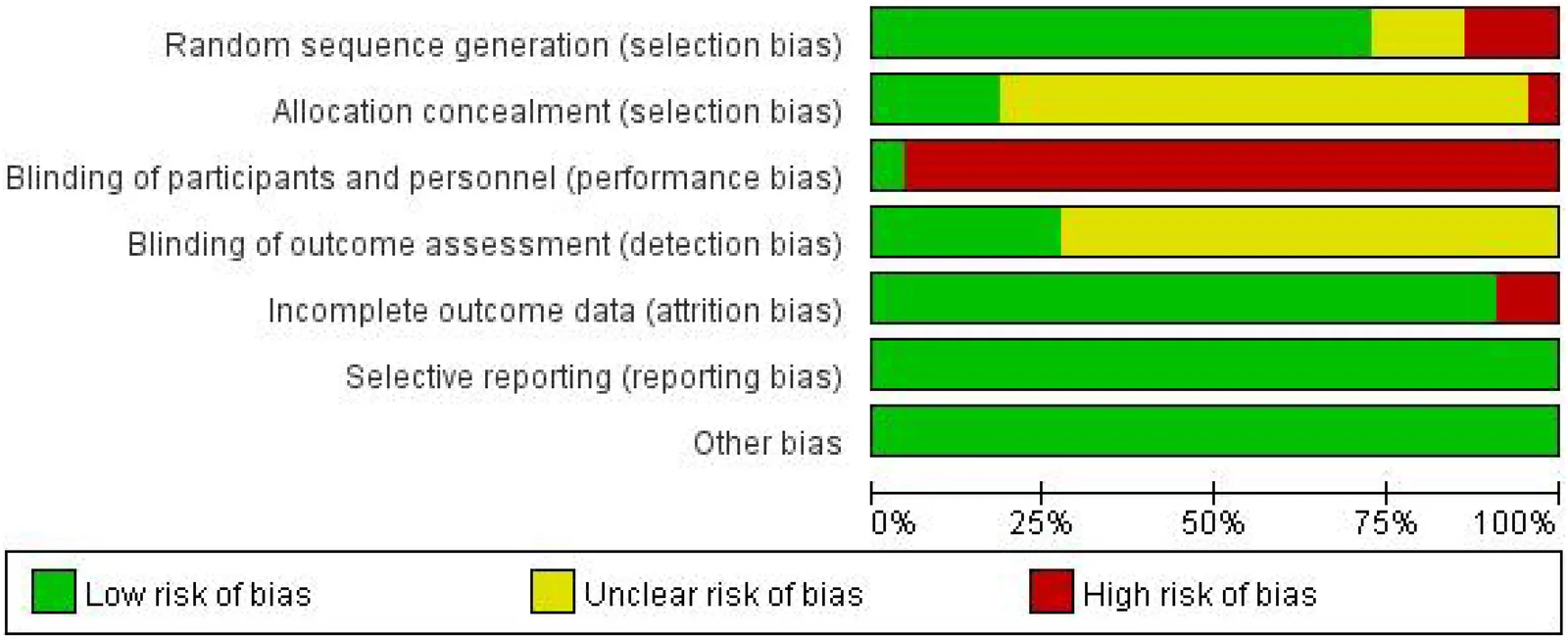
Bias risk of the included studies.

**Figure 3:**
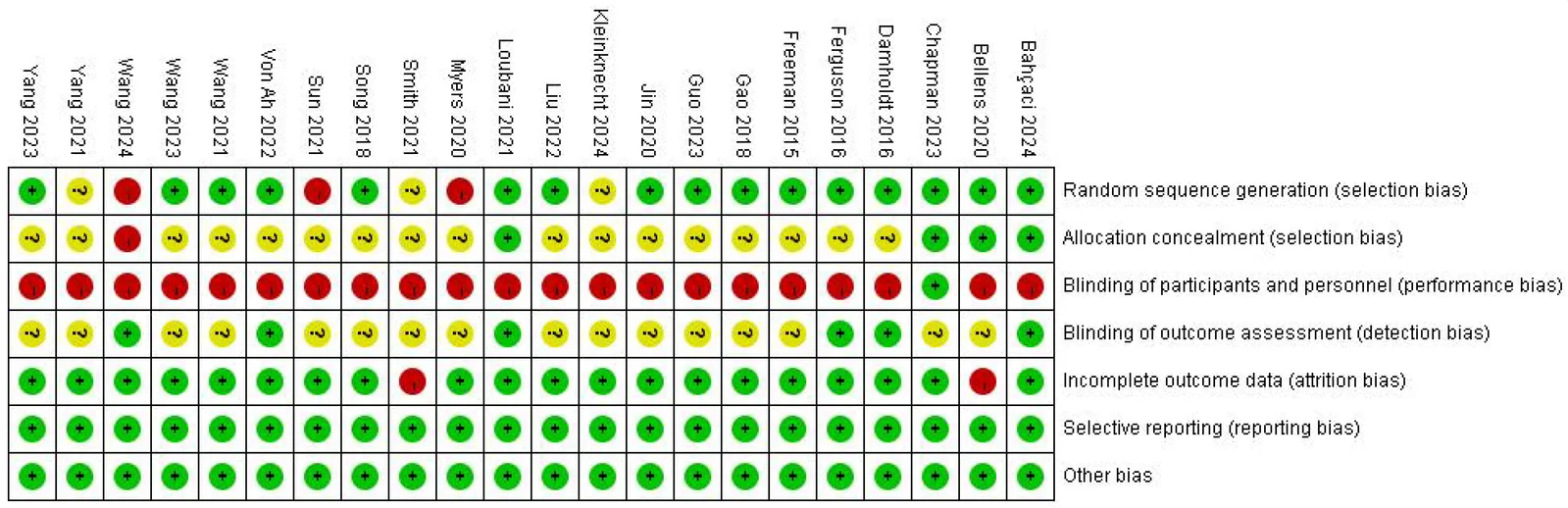
Bias risk summary of the included studies.

### Traditional pairwise meta-analysis and subgroup analysis

#### Subjective cognitive function

Fourteen studies (14, 16, 17, 21, 22, 25–27, 29, 31, 33–36) involving 807 participants assessed subjective cognitive outcomes. The pooled analysis using a random-effects model demonstrated a significant effect in favour of the experimental group (SMD = 0.57, 95% CI 0.23 to 0.91, P = 0.001), although substantial heterogeneity was observed across studies (I² = 81)%, P < 0.00001). Subgroup analysis by intervention type indicated significant differences among subgroups (P < 0.0001). Significant effects were identified for DPBI (SMD = 0.65, 95% CI 0.37 to 0.94, P < 0.00001; I² = 0%), DEI (SMD = 2.53, 95% CI 1.79 to 3.27, P < 0.00001), and DMI (SMD = 0.61, 95% CI 0.22 to 0.99, P = 0.002). No significant effects were found for DCT (SMD = 0.27, 95% CI −0.28 to 0.83, P = 0.33; I² = 81%) or DRI (SMD = 0.41, 95% CI −0.01 to 0.84, P = 0.06; I² = 0%)(**Supplementary Figure S1**).

#### Objective cognitive function

Eleven studies (18–21, 23, 24, 26, 28, 30–32) involving 843 participants assessed objective cognitive outcomes. The random-effects meta-analysis showed a significant overall effect in favour of the experimental group (SMD = 1.95, 95% CI 1.24 to 2.67, P < 0.00001). However, substantial between-study heterogeneity was observed (I² = 95%, P < 0.00001). Subgroup analysis according to intervention type revealed significant differences among subgroups (P = 0.0009). Significant effects favouring the experimental group were observed for DRI (SMD = 2.59, 95% CI 1.29 to 3.88, P < 0.0001; I² = 96%), DEI (SMD = 1.48, 95% CI 0.86 to 2.10, P < 0.00001), and DMI (SMD = 1.51, 95% CI 0.67 to 2.34, P = 0.0004; I² = 89%). In contrast, no significant between-group difference was observed for DCT (SMD = 0.21, 95% CI −0.37 to 0.79, P = 0.48)(**Supplementary Figure S2**).

### Network geometry and inconsistency assessment

#### Subjective cognitive function

Fourteen studies reporting subjective cognitive function outcomes were included, involving six intervention nodes: CON, DCT, DRI, DPBI, DEI, and DMI. The evidence network exhibited a star-shaped structure centered on CON, with all digital interventions directly compared only with conventional care. No head-to-head comparisons among active interventions were identified, and no closed loops were formed in the network. Therefore, inconsistency assessment was not applicable (**Figure 4**).

**Figure 4:**
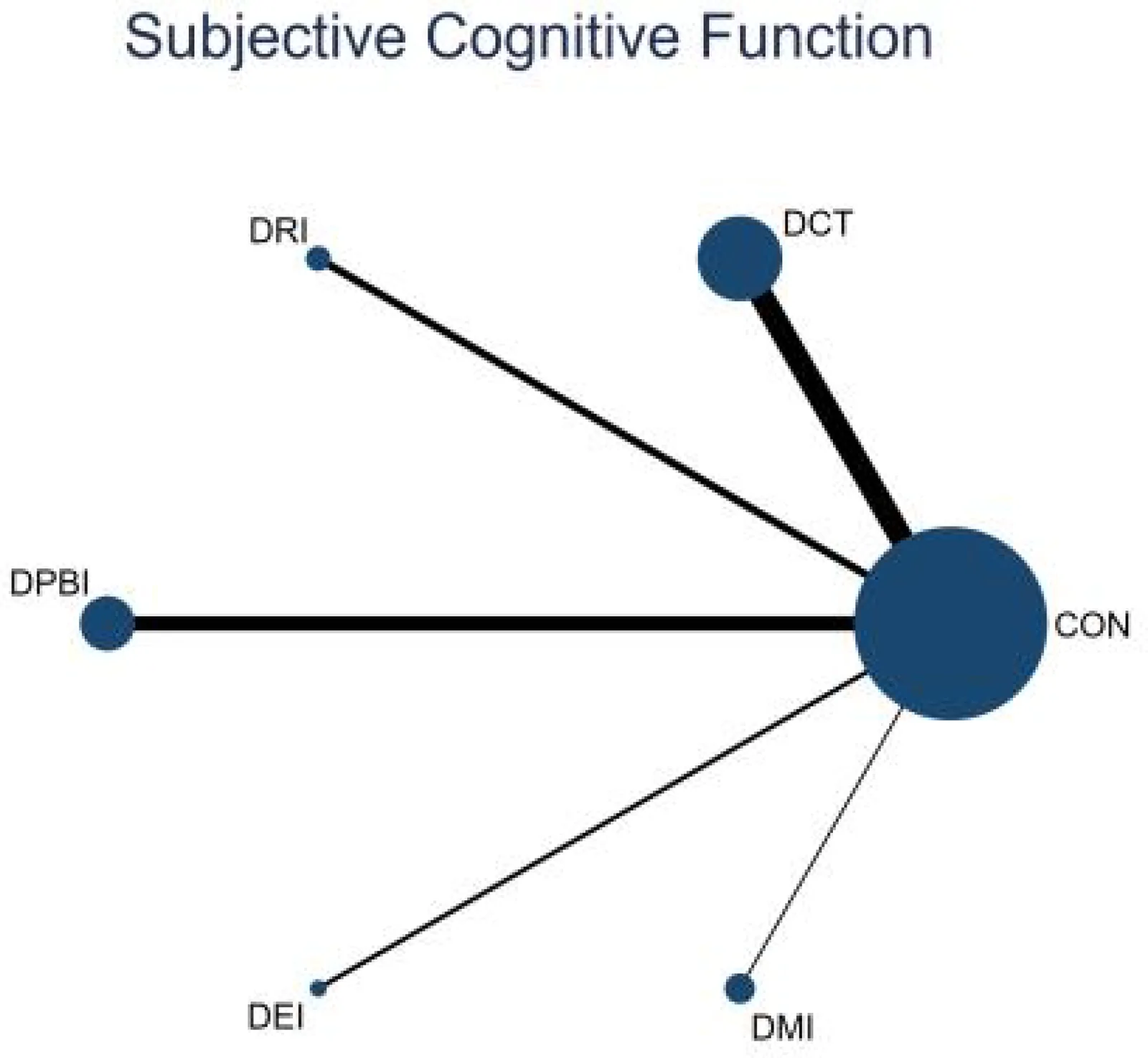
Network diagram of subjective cognition.

#### Objective cognitive function

Eleven studies reporting objective cognitive function outcomes were included, involving five intervention nodes: CON, DCT, DRI, DEI, and DMI. The evidence network showed a star-shaped configuration centered on CON, with all digital interventions being directly compared only with conventional care. No direct comparisons between active interventions were available, and no closed loops were identified. Therefore, inconsistency assessment was not applicable (**Figure 5**).

**Figure 5:**
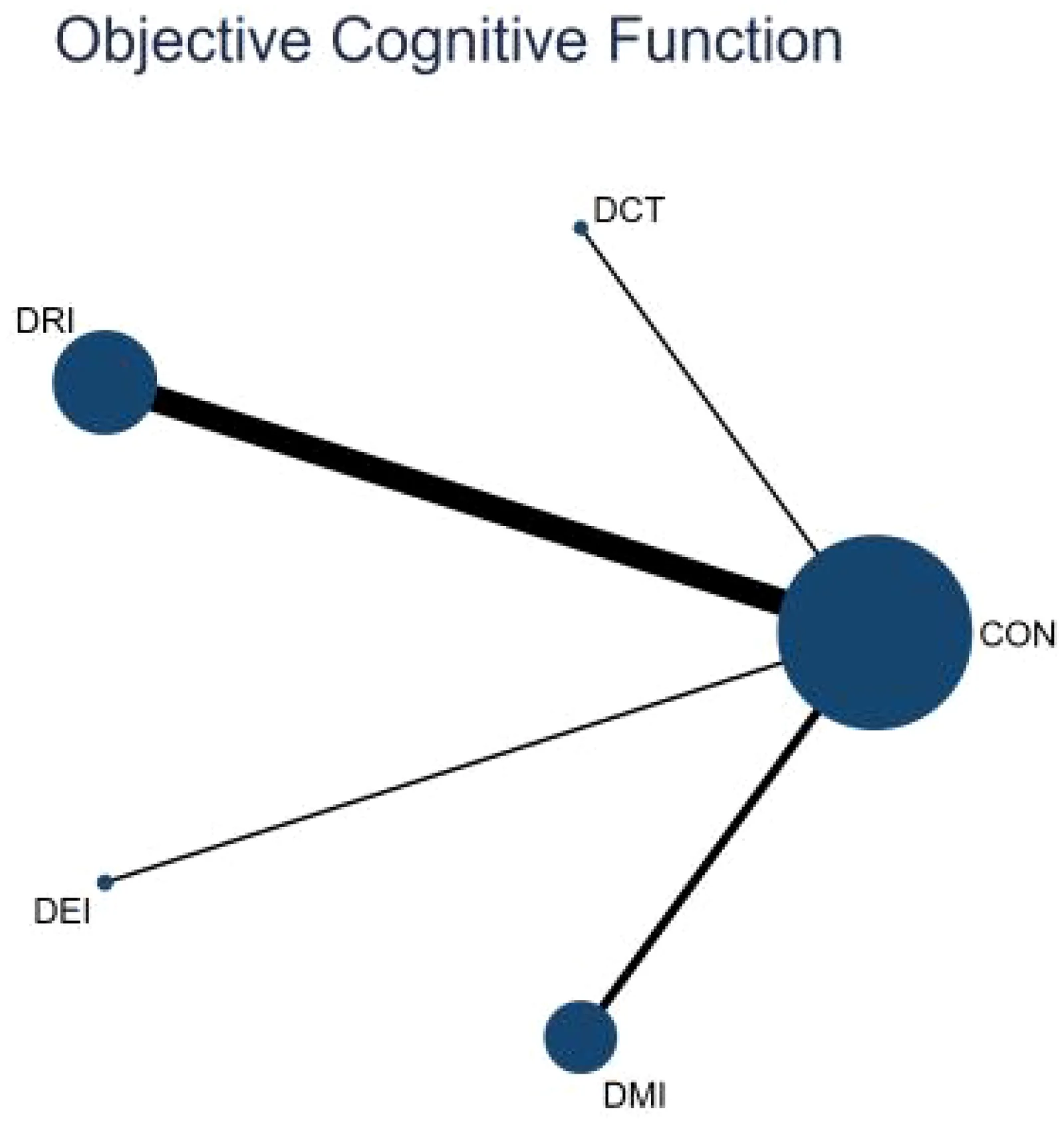
Network diagram of objective cognition. CON, control group; DCT, digital cognitive training; DRI, digital rehabilitation intervention; DMI, digital multimodal intervention; DPBI, digital psychological and behavioral intervention; DEI, digital exercise intervention.

### Network meta-analysis

#### Subjective cognitive function

The network meta-analysis showed that, compared with CON, DEI was associated with a significant improvement in subjective cognitive function (SMD = 2.53, 95% CI 1.58 to 3.48). A significant effect was also observed for DPBI (SMD = 0.44, 95% CI 0.03 to 0.85). Based on the network estimates, DEI demonstrated significantly greater effects than DMI (SMD = 1.92, 95% CI 0.74 to 3.11), DPBI (SMD = 2.09, 95% CI 1.06 to 3.12), DRI (SMD = 2.09, 95% CI 0.97 to 3.22), and DCT (SMD = 2.40, 95% CI 1.39 to 3.41). No statistically significant differences were observed among the remaining active interventions. Detailed pairwise comparisons are presented in **Table 2**.

**Table 2:** Network meta-analysis results of digital interventions for improving subjective cognitive function in breast cancer patients treated with chemotherapy [SMD (95% CI)].

Interventions	DEI	DMI	DPBI	DRI	DCT
DMI	1.92 (0.74, 3.11)	–	–	–	–
DPBI	2.09 (1.06, 3.12)	0.17 (-0.65, 0.98)	–	–	–
DRI	2.09 (0.97, 3.22)	0.17 (-0.76, 1.10)	0.00 (-0.73, 0.73)	–	–
DCT	2.40 (1.39, 3.41)	0.48 (-0.30, 1.27)	0.31 (-0.22, 0.85)	0.31 (-0.38, 1.00)	–
CON	2.53 (1.58, 3.48)	0.61 (-0.10, 1.31)	0.44 (0.03, 0.85)	0.44 (-0.17, 1.04)	0.13 (-0.22, 0.47)

Values represent the effect of the row intervention relative to the column intervention; positive SMDs favor the row intervention.

Based on SUCRA values, DEI showed the highest ranking probability for improving subjective cognitive function (SUCRA = 100.0%), followed by DMI (62.9%), DPBI (53.5%), DRI (51.5%), DCT (24.2%), and CON (7.5%). Details of the cumulative ranking probability plot are shown in **Figure 6**.

**Figure 6:**
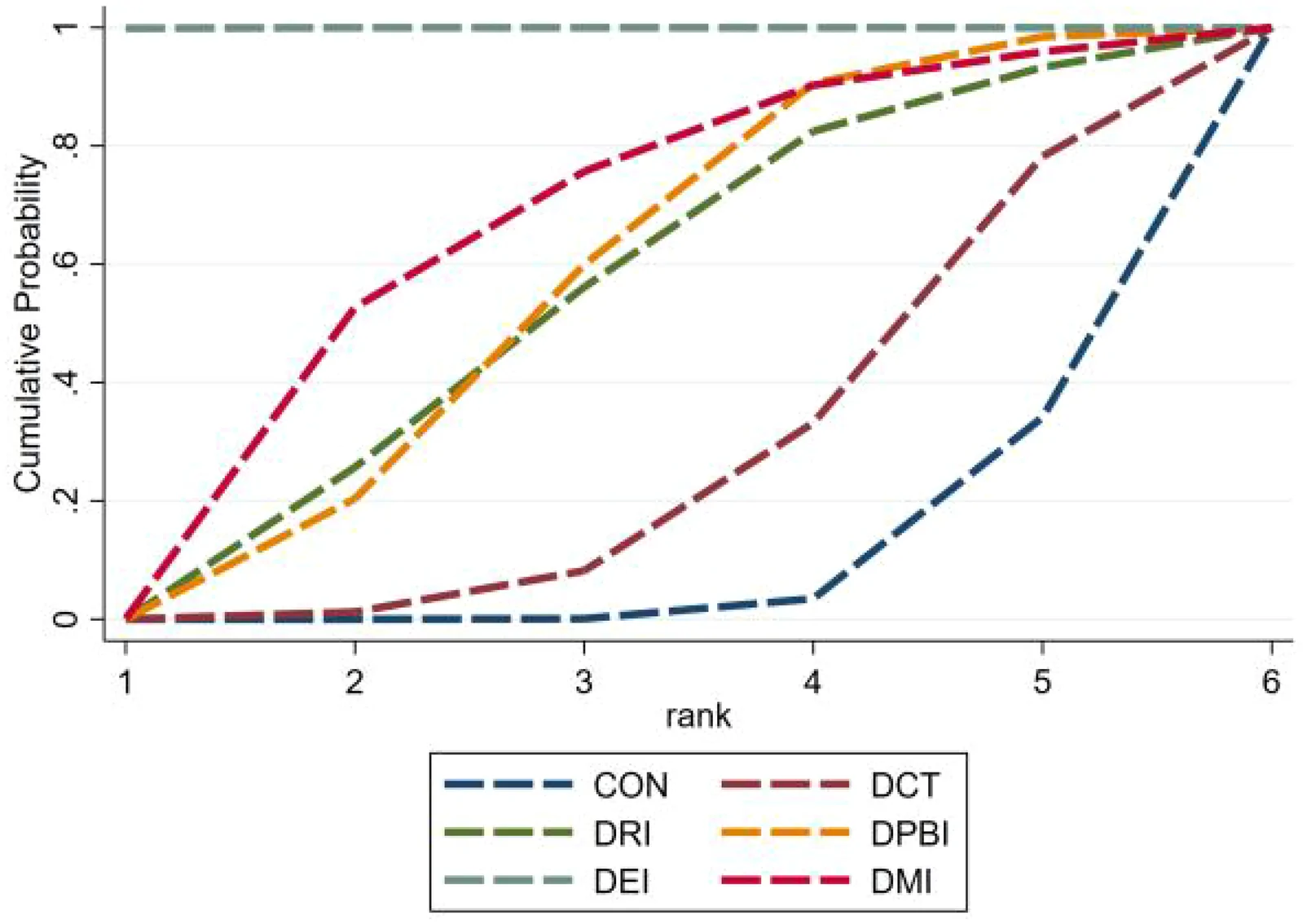
Cumulative ranking plot of subjective cognition.

#### Objective cognitive function

The network meta-analysis showed that, compared with CON, DRI significantly improved objective cognitive function (SMD = 2.59, 95% CI 1.17 to 4.02). No statistically significant differences were observed in the remaining comparisons among interventions. Detailed results are presented in **Table 3**.

**Table 3:** Network meta-analysis results of digital interventions for improving objective cognitive function in breast cancer patients treated with chemotherapy [SMD (95% CI)].

Interventions	DRI	DMI	DEI	DCT
DMI	1.03 (-1.42, 3.49)	–	–	–
DEI	1.11 (-2.65, 4.87)	0.08 (-3.93, 4.09)	–	–
DCT	2.39 (-1.36, 6.14)	1.35 (-2.65, 5.36)	1.28 (-3.64, 6.19)	–
CON	2.59 (1.17, 4.02)	1.56 (-0.43, 3.56)	1.48 (-1.99, 4.96)	0.21 (-3.26, 3.68)

Values represent the effect of the row intervention relative to the column intervention; positive SMDs favor the row intervention.

According to SUCRA probabilities, DRI ranked highest for improving objective cognitive function (SUCRA = 85.5%), followed by DMI (60.5%), DEI (55.8%), DCT (30.3%), and CON (17.9%). Details of the cumulative ranking probability plot are shown in **Figure 7**. These findings suggest that DRI showed the highest probability of being ranked first within the current evidence network; however, the ranking results should be interpreted cautiously considering the limited number of available studies and the uncertainty of comparative estimates.

**Figure 7:**
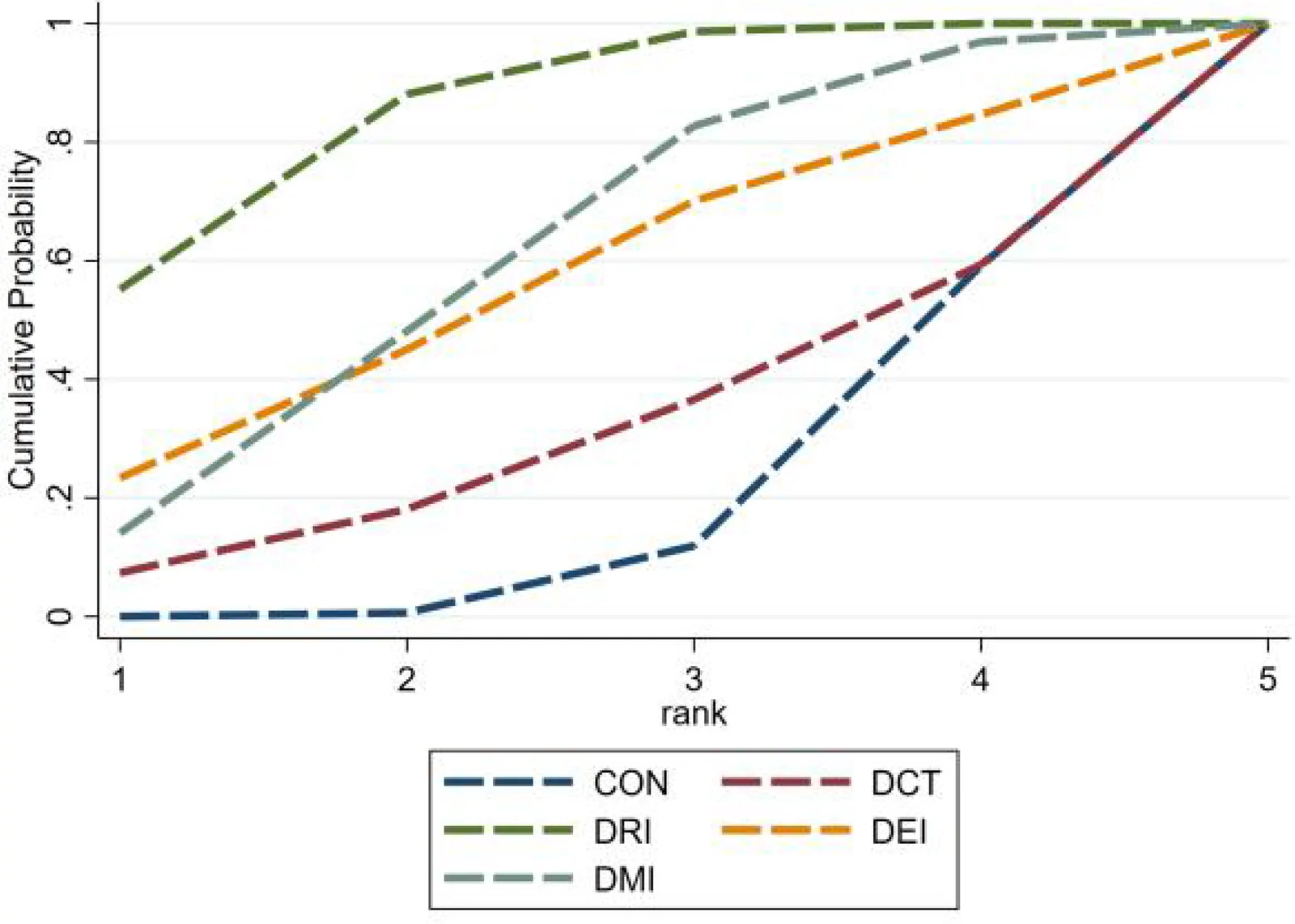
Cumulative ranking plot of objective cognition.

### Publication bias test

Comparison-adjusted funnel plots were used to assess potential publication bias and small-study effects for both subjective and objective cognitive outcomes. The plots were generally symmetrical overall, although a few studies fell outside the funnel area, suggesting the possible presence of publication bias or small-study effects. The funnel plots are shown in **Figure 8**.

**Figure 8:**
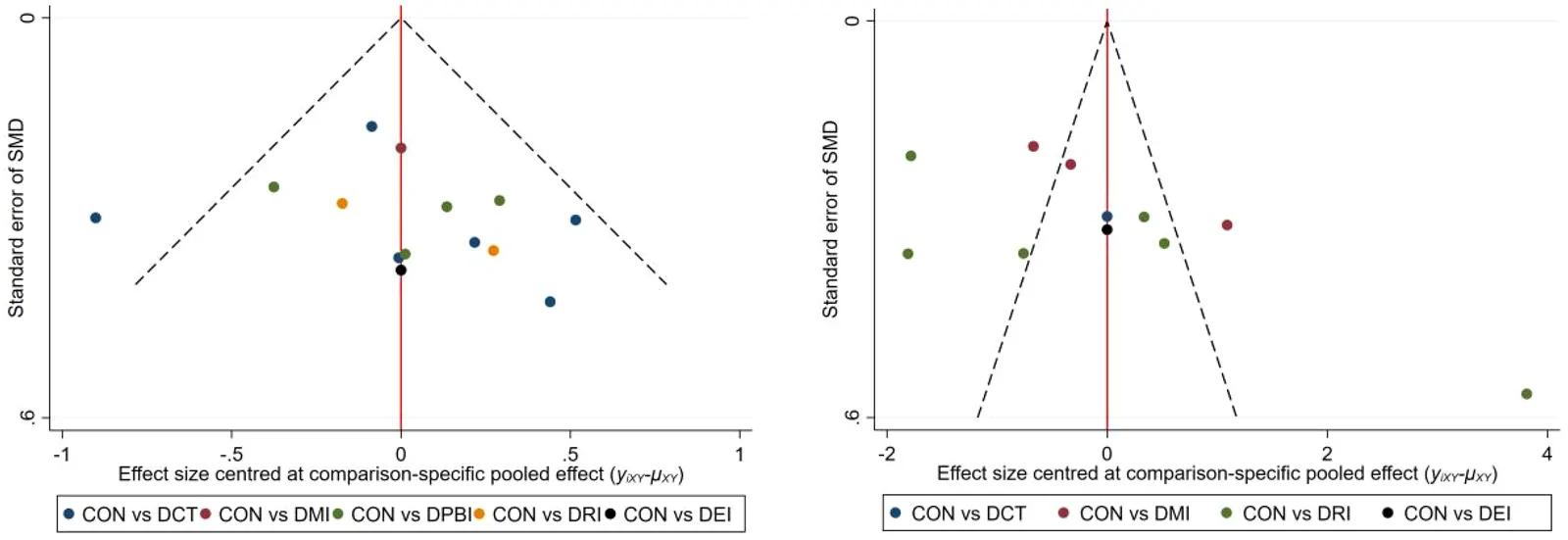
Comparison-adjusted funnel plots of subjective cognition and objective cognition.

### Sensitivity analysis

Network-specific leave-one-out sensitivity analyses were performed to assess the robustness of the NMA findings. For subjective cognitive function, DEI remained the highest-ranked intervention in all analyses in which the DEI node remained estimable, whereas the statistical significance of DPBI versus CON was lost after exclusion of several individual studies (**Supplementary Table S2**). For objective cognitive function, the significant effect of DRI versus CON was maintained after sequential exclusion of each study, and DRI consistently ranked first. Exclusion of Gao et al. resulted in the largest reduction in between-study heterogeneity (τ² from 3.052 to 1.027), without changing the statistical significance of DRI versus CON (**Supplementary Table S3**). In the fixed-effect sensitivity analyses, the overall direction of effects was generally consistent with that of the random-effects analyses; however, DMI versus CON became statistically significant for subjective cognitive function, whereas DMI and DEI versus CON became statistically significant for objective cognitive function. Given the substantial heterogeneity across studies, the random-effects model was retained as the primary analysis (**Supplementary Table S4**).

### Certainty of evidence

According to the GRADE approach, the overall certainty of evidence regarding the effects of digital health interventions on both subjective and objective cognitive function in breast cancer patients was rated as “low” (**Table 4**). The primary reasons for downgrading the certainty of evidence included serious risk of bias (RoB) and substantial heterogeneity within the pooled effect estimates.

**Table 4:** GRADE summary of evidence for digital health interventions.

Certainty assessment								Effect	Certainty	Importance
Outcome measures	No. of studies	Study design	Risk of bias	Inconsistency	Indirectness	Imprecision	Other considerations	SMD (95% CI)		
Subjective cognitive function	14	randomised trials	Serious*	Serious^	not serious	not serious	none	0.57(0.23, 0.91)	⊕⊕○○ Low	CRITICAL
Objective cognitive function	11	randomised trials	Serious*	Serious^	not serious	not serious	none	1.95(1.24, 2.67)	⊕⊕○○ Low	CRITICAL

*Downgraded by one level for risk of bias:Most included trials were at high risk of performance bias due to the lack of blinding of participants and personnel.; ^Downgraded by one level for inconsistency due to high statistical heterogeneity (I^2^>50%).

## Discussion

This network meta-analysis compared five categories of digital health interventions for cognitive function in breast cancer patients treated with chemotherapy. The findings differed according to the cognitive outcome assessed. DEI and DPBI were associated with improvements in subjective cognitive function compared with CON, whereas DRI was the only intervention showing a statistically significant benefit for objective cognitive function. Although these results point to potentially different roles for digital interventions, their interpretation is limited by the heterogeneity of the trials and the low certainty of the evidence.

For subjective cognitive function, the network meta-analysis indicated that DEI demonstrated the most favorable effect among the included digital interventions and ranked first in the SUCRA analysis (SUCRA = 100.0%), suggesting that it may be a particularly promising approach for improving patients’ perceived cognitive function.

Subjective cognition primarily reflects patients’ self-perceived cognitive abilities. Variations in this domain are not only associated with objective cognitive performance but are also highly susceptible to factors such as emotional state, fatigue severity, sleep quality, and psychological adaptation (37, 38). Breast cancer patients treated with chemotherapy frequently experience concomitant anxiety, depression, and cancer-related fatigue, which may exacerbate their subjective perception of cognitive decline (39, 40). By leveraging digital platforms to guide patients in engaging in regular physical activity, DEI not only improves physical fitness but also helps alleviate negative emotions and fatigue, thereby indirectly enhancing patients’ subjective appraisal of their cognitive status (41). In addition, digitally delivered exercise interventions may reduce barriers to participation and provide greater flexibility and interactivity. Remote supervision, feedback, and ongoing support from healthcare professionals may further enhance patients’ engagement and self-management capacity, which may contribute to improvements in perceived cognitive function.

Moreover, physical exercise intrinsically exerts favorable stimulatory and regulatory effects on the nervous system. Previous studies (42, 43) have suggested that exercise may mitigate chemotherapy-related neurofunctional impairment by improving cerebral blood flow, promoting neurotransmitter release, and enhancing neuroplasticity. These mechanisms may contribute not only to improvements in cognitive function but also to more favorable perceptions of cognitive ability. The observed advantage of DEI in the present analysis is also broadly consistent with previous evidence suggesting that exercise-based interventions may be effective non-pharmacological approaches for improving perceived cognitive function in breast cancer patients (44). Clinically, these findings support the potential incorporation of digitally delivered exercise programs into survivorship and supportive care, particularly for patients with prominent subjective cognitive complaints, fatigue, or emotional symptoms. Nevertheless, intervention design should consider patients’ physical condition, treatment stage, symptom burden, and digital literacy to optimize feasibility, adherence, and potential benefit.

For objective cognitive function, the present network meta-analysis showed that DRI was the only intervention associated with a statistically significant improvement compared with CON and had the highest SUCRA value. However, DRI did not differ significantly from the other active digital interventions, and its apparent advantage should therefore be interpreted cautiously.

Unlike subjective cognition, objective cognitive function is assessed using standardized neuropsychological measures that capture performance in specific domains such as memory, executive function, and processing speed (45). The favorable effect observed for DRI may be related primarily to its rehabilitation-oriented content. Cognitive rehabilitation commonly incorporates structured cognitive training, compensatory strategies, and repeated practice targeting specific deficits. Previous reviews in cancer survivors have reported improvements in at least some objectively measured cognitive outcomes following cognitive rehabilitation, although the magnitude of benefit and the cognitive domains affected have varied across studies (46, 47). This evidence provides a plausible basis for the observed association between DRI and objective cognitive improvement.

As with DEI, digital delivery may enhance the accessibility and continuity of rehabilitation by allowing structured interventions to be provided beyond conventional face-to-face settings (48, 49). However, the digital platform itself should not be assumed to account for the cognitive benefit. Rather, its principal contribution may lie in facilitating the delivery of structured rehabilitation, repeated practice, and ongoing professional support. The cognitive effects of DRI are therefore more plausibly attributable to the rehabilitation content, with digital technology serving as a means of improving access and implementation.

Despite the significant effect observed compared with CON, the evidence supporting DRI remains limited. No statistically significant differences were found between DRI and the other active interventions, and several network estimates had wide confidence intervals. In addition, previous rehabilitation studies have shown considerable variation in intervention content, intensity, duration, cognitive assessment methods, and participant characteristics (46–49). Accordingly, DRI should be regarded as a promising approach for improving objectively measured cognitive function rather than as an intervention with established superiority. Further adequately powered randomized controlled trials using standardized neuropsychological assessments, clearly defined rehabilitation protocols, and longer follow-up periods are needed to confirm the robustness of these findings.

Taken together, the present findings suggest that different types of digital interventions may have distinct strengths across cognitive dimensions. For subjective cognitive function, DEI showed the largest estimated effect and the highest SUCRA ranking, while DPBI also demonstrated a significant benefit compared with CON. However, the evidence supporting DEI was based on a limited number of studies, and comparisons between active interventions were primarily informed by indirect evidence; therefore, its apparent advantage should be interpreted cautiously. For objective cognitive function, DRI showed the most favorable effect compared with CON and ranked highest in the SUCRA analysis. This divergence may reflect differences between subjective and objective cognitive outcomes: subjective cognitive complaints are more closely influenced by fatigue, emotional symptoms, sleep, and self-perception, whereas objective outcomes reflect performance on standardized cognitive tasks. Therefore, the present results do not support the assumption that a single digital intervention is uniformly optimal across both subjective and objective cognitive outcomes. Instead, intervention selection may need to be guided by the predominant cognitive complaint, associated symptom burden, rehabilitation goals, and patient characteristics.

From a nursing and supportive care standpoint, these results reinforce the potential value of digital health approaches in long-term cancer rehabilitation. Digital interventions may help integrate cognitive care into routine survivorship management by enabling remote supervision, individualized feedback, and repeated practice in everyday environments. This may be especially beneficial for patients who have limited access to center-based rehabilitation or who require sustained support after active treatment. Moreover, digital modalities may facilitate more flexible, patient-centered care models and improve the reach of non-pharmacological interventions in oncology settings. Future implementation efforts should therefore consider not only efficacy, but also usability, accessibility, cost, patient preference, and integration into existing supportive care services.

## Study limitations

Several limitations of the present study should be acknowledged. First, owing to the inherent nature of digital health interventions, blinding of participants and personnel was largely unfeasible across the primary trials, inevitably introducing a potential risk of performance bias. Second, the limited number of included studies available for certain intervention nodes may compromise the statistical power and robustness of the corresponding pooled estimates. Third, the majority of the analyzed trials predominantly focused on short-term outcomes; thus, there remains a dearth of evidence regarding the long-term sustainability of the observed cognitive improvements. Finally, the included intervention programs in the study differed in terms of form, frequency and duration, which might have had some impact on the results.

## Conclusion

This network meta-analysis suggests that digital health interventions may improve cognitive function in breast cancer patients treated with chemotherapy. DEI showed the greatest potential for improving subjective cognitive function, whereas DRI appeared more promising for objective cognitive function. However, given the low certainty of evidence and the limited evidence available for some intervention categories, these findings should be interpreted cautiously and confirmed by further high-quality randomized controlled trials.

## Data Availability

The original contributions presented in the study are included in the article/**Supplementary Material**. Further inquiries can be directed to the corresponding author.
