# Supplementary material for: Digital health interventions for cognitive function in breast cancer patients treated with chemotherapy: a network meta-analysis of subjective and objective cognitive outcomes
Source: Front Oncol. 2026 Oct 29;16:1989710. doi: 10.3389/fonc.2026.1989710 (PMC13754666; doi:10.3389/fonc.2026.1989710)
Supplement: Supplementary file 1 [file DataSheet1.docx]

**Supplementary materials**

Figure S1. Results of traditional meta-analyses on subjective cognition

Figure S2. Results of traditional meta-analyses on objective cognition

Table S1. Search strategy

| **Database** | **SearchStep** | **SearchTerms** |
| --- | --- | --- |
| PubMed | #1 | ("Breast Neoplasms"[Mesh] OR breast neoplas*[tiab] OR breast cancer*[tiab] OR breast tumor*[tiab] OR breast carcinoma*[tiab] OR mammary cancer*[tiab] OR mammary neoplas*[tiab]) |
|  | #2 | ("Cognition"[Mesh] OR "Cognitive Dysfunction"[Mesh] OR "Neurocognitive Disorders"[Mesh] OR cogniti*[tiab] OR memory[tiab] OR executive function[tiab] OR attention[tiab] OR cognitive fatigue[tiab] OR chemo brain[tiab] OR chemobrain[tiab] OR chemo fog[tiab] OR CRCI[tiab] OR cancer-related cognitive impair*[tiab]) |
|  | #3 | ("Exercise"[Mesh] OR "Exercise Therapy"[Mesh] OR "Physical Therapy Modalities"[Mesh] OR exercise*[tiab] OR aerobic[tiab] OR resistance training[tiab] OR resistance exercise[tiab] OR multicomponent exercise[tiab] OR mind-body[tiab] OR physical activity[tiab] OR Tai Chi[tiab] OR yoga[tiab] OR qigong[tiab] OR walking[tiab] OR cycling[tiab] OR strength training[tiab]) |
|  | #4 | #1 AND #2 AND #3 |
| Embase | #1 | 'breast cancer'/exp OR 'breast tumor'/exp OR 'breast carcinoma'/exp OR 'breast neoplas*':ti,ab,kw OR 'breast cancer*':ti,ab,kw OR 'breast tumor*':ti,ab,kw OR 'mammary cancer':ti,ab,kw |
|  | #2 | 'cognition'/exp OR 'cognitive defect'/exp OR 'memory'/exp OR 'executive function'/exp OR 'attention'/exp OR 'mental fatigue'/exp OR cogniti*:ti,ab,kw OR memory:ti,ab,kw OR 'executive function':ti,ab,kw OR attention:ti,ab,kw OR 'cognitive fatigue':ti,ab,kw OR 'chemo brain':ti,ab,kw OR chemobrain:ti,ab,kw OR crci:ti,ab,kw OR 'cancer-related cognitive impair*':ti,ab,kw |
|  | #3 | 'exercise'/exp OR 'exercise therapy'/exp OR 'physical activity'/exp OR 'aerobic exercise'/exp OR 'resistance training'/exp OR 'mind body exercise'/exp OR exercise*:ti,ab,kw OR aerobic:ti,ab,kw OR 'resistance training':ti,ab,kw OR 'resistance exercise':ti,ab,kw OR 'multicomponent exercise':ti,ab,kw OR 'mind-body':ti,ab,kw OR 'physical activity':ti,ab,kw OR 'tai chi':ti,ab,kw OR yoga:ti,ab,kw OR qigong:ti,ab,kw OR walking:ti,ab,kw OR cycling:ti,ab,kw OR 'strength training':ti,ab,kw |
|  | #4 | #1 AND #2 AND #3 |
| The Cochrane Library | #1 | MeSH descriptor: [Breast Neoplasms] explode all trees OR (breast NEXT neoplas*):ti,ab,kw OR (breast NEXT cancer*):ti,ab,kw OR (breast NEXT tumor*):ti,ab,kw OR "mammary cancer":ti,ab,kw |
|  | #2 | MeSH descriptor: [Cognition] explode all trees OR MeSH descriptor: [Cognitive Dysfunction] explode all trees OR MeSH descriptor: [Memory] explode all trees OR MeSH descriptor: [Executive Function] explode all trees OR MeSH descriptor: [Attention] explode all trees OR cogniti*:ti,ab,kw OR memory:ti,ab,kw OR "executive function":ti,ab,kw OR attention:ti,ab,kw OR "cognitive fatigue":ti,ab,kw OR "chemo brain":ti,ab,kw OR chemobrain:ti,ab,kw OR crci:ti,ab,kw OR "cancer-related cognitive impair*":ti,ab,kw |
|  | #3 | MeSH descriptor: [Exercise] explode all trees OR MeSH descriptor: [Exercise Therapy] explode all trees OR MeSH descriptor: [Physical Therapy Modalities] explode all trees OR exercise*:ti,ab,kw OR aerobic:ti,ab,kw OR "resistance training":ti,ab,kw OR "resistance exercise":ti,ab,kw OR "multicomponent exercise":ti,ab,kw OR "mind-body":ti,ab,kw OR "physical activity":ti,ab,kw OR "tai chi":ti,ab,kw OR yoga:ti,ab,kw OR qigong:ti,ab,kw OR walking:ti,ab,kw OR cycling:ti,ab,kw OR "strength training":ti,ab,kw |
|  | #4 | #1 AND #2 AND #3 |
| Web of Science | #1 | TS=("breast neoplas*" OR "breast cancer*" OR "breast tumor*" OR "breast carcinoma*" OR "mammary cancer*") |
|  | #2 | TS=("cogniti*" OR "cognitive dysfunction" OR "cognitive impair*" OR "cognitive decline" OR memory OR "executive function" OR attention OR "cognitive fatigue" OR "chemo brain" OR chemobrain OR "chemo fog" OR CRCI OR "cancer-related cognitive impair*") |
|  | #3 | TS=("exercise*" OR aerobic OR "resistance training" OR "resistance exercise" OR "multicomponent exercise" OR "mind-body" OR "physical activity" OR "tai chi" OR yoga OR qigong OR walking OR cycling OR "strength training") |
|  | #4 | #1 AND #2 AND #3 |
| CNKI | #1 | SU=('乳腺癌'+'乳癌'+'乳腺肿瘤'+'乳腺恶性肿瘤') |
|  | #2 | SU=('认知功能'+'认知障碍'+'认知功能减退'+'记忆力'+'注意力'+'执行功能'+'认知疲劳'+'化疗脑'+'癌症相关认知障碍') |
|  | #3 | SU=('运动'+'有氧运动'+'抗阻运动'+'力量训练'+'多成分运动'+'身心运动'+'体育锻炼'+'运动干预'+'太极'+'瑜伽'+'气功'+'步行'+'骑车') |
|  | #4 | #1 AND #2 AND #3 |
| Wanfang Data | #1 | (主题:(乳腺癌 OR 乳癌 OR 乳腺肿瘤 OR 乳腺恶性肿瘤)) |
|  | #2 | (主题:(认知功能 OR 认知障碍 OR 认知功能减退 OR 记忆力 OR 注意力 OR 执行功能 OR 认知疲劳 OR 化疗脑 OR 癌症相关认知障碍)) |
|  | #3 | (主题:(运动 OR 有氧运动 OR 抗阻运动 OR 力量训练 OR 多成分运动 OR 身心运动 OR 体育锻炼 OR 运动干预 OR 太极 OR 瑜伽 OR 气功 OR 步行 OR 骑车)) |
|  | #4 | #1 AND #2 AND #3 |
| VIP | #1 | (T=(乳腺癌 OR 乳癌 OR 乳腺肿瘤 OR 乳腺恶性肿瘤)) |
|  | #2 | (T=(认知功能 OR 认知障碍 OR 认知功能减退 OR 记忆力 OR 注意力 OR 执行功能 OR 认知疲劳 OR 化疗脑 OR 癌症相关认知障碍)) |
|  | #3 | (T=(运动 OR 有氧运动 OR 抗阻运动 OR 力量训练 OR 多成分运动 OR 身心运动 OR 体育锻炼 OR 运动干预 OR 太极 OR 瑜伽 OR 气功 OR 步行 OR 骑车)) |
|  | #4 | #1 AND #2 AND #3 |
| SinoMed | #1 | "乳腺肿瘤"[中文主题词:智能] OR "乳腺癌"[扩展:智能] OR "乳癌" OR "乳腺恶性肿瘤" |
|  | #2 | "认知障碍"[中文主题词:智能] OR "轻度认知障碍"[中文主题词:智能] OR "记忆障碍"[中文主题词:智能] OR "认知功能减退" OR "认知功能障碍" OR "认知功能下降" OR "记忆力下降" OR "注意力下降" OR "执行功能" OR "认知疲劳" OR "化疗脑" OR "癌症相关认知障碍" |
|  | #3 | "运动"[中文主题词:智能] OR "运动疗法"[中文主题词:智能] OR "体育锻炼"[中文主题词:智能] OR "有氧运动" OR "抗阻运动" OR "力量训练" OR "多成分运动" OR "身心运动" OR "太极" OR "瑜伽" OR "气功" OR "步行" OR "骑车" |
|  | #4 | #1 AND #2 AND #3 |

Table S2. Network-specific leave-one-out sensitivity analysis for subjective cognitive function

| **Excluded study** | **τ2** | **DEI vs CON, SMD (95% CI)** | **DPBI vs CON, SMD (95% CI)** | **DEI rank** | **Main finding** |
| --- | --- | --- | --- | --- | --- |
| None | 0.0918 | 2.53 (1.58, 3.47) | 0.44 (0.03, 0.85) | 1 | Original analysis |
| Guo 2023 | 0.0918 | NE | 0.44 (0.03, 0.85) | NE | DEI node removed |
| Wang 2021 | 0.0994 | 2.53 (1.57, 3.49) | 0.34 (−0.15, 0.83) | 1 | DPBI no longer significant |
| Chapman 2023 | 0.0217 | 2.53 (1.74, 3.32) | 0.43 (0.12, 0.75) | 1 | Marked reduction in heterogeneity |
| Kleinknecht 2024 | 0.1119 | 2.53 (1.54, 3.51) | 0.44 (0.01, 0.87) | 1 | Main finding unchanged |
| Bahçaci 2024 | 0.1131 | 2.53 (1.54, 3.52) | 0.40 (−0.11, 0.90) | 1 | DPBI no longer significant |
| Freeman 2015 | 0.0818 | 2.53 (1.60, 3.45) | 0.59 (0.12, 1.06) | 1 | Main finding unchanged |
| Sun 2021 | 0.0918 | 2.53 (1.58, 3.47) | 0.44 (0.03, 0.85) | 1 | DMI node removed |
| Ferguson 2016 | 0.1126 | 2.53 (1.54, 3.52) | 0.44 (−0.05, 0.93) | 1 | DPBI no longer significant |
| Smith 2021 | 0.0936 | 2.53 (1.58, 3.48) | 0.44 (0.03, 0.85) | 1 | Main finding unchanged |
| Myers 2020 | 0.1024 | 2.53 (1.56, 3.50) | 0.44 (0.02, 0.86) | 1 | Main finding unchanged |
| Von Ah 2022 | 0.1055 | 2.53 (1.56, 3.50) | 0.44 (0.01, 0.87) | 1 | Main finding unchanged |
| Damholdt 2016 | 0.1219 | 2.53(1.52,3.53) | 0.44(-0.00,0.88) | 1 | DPBI became borderline/non-significant |
| Bellens 2020 | 0.0682 | 2.53(1.63,3.43) | 0.44(0.06,0.82) | 1 | Main finding unchanged |
| Loubani 2021 | 0.1024 | 2.53(1.56,3.50) | 0.44(0.02,0.86) | 1 | Main finding unchanged |

Table S3. Network-specific leave-one-out sensitivity analysis for objective cognitive function

| **Excluded study** | **τ2** | **DRI·vs·CON,SMD·(95% CI)** | **DRI·rank** | **Main finding** |
| --- | --- | --- | --- | --- |
| None | 3.052 | 2.60·(1.17,4.02) | 1 | Original analysis |
| Guo·2023 | 3.052 | 2.60·(1.17,4.02) | 1 | DEI·node ·removed;DRI·unchanged |
| Wang·2023 | 3.495 | 2.60·(1.08,4.12) | 1 | Main finding ·unchanged |
| Liu·2022 | 3.592 | 2.60·(1.06,4.15) | 1 | Main finding ·unchanged |
| Gao·2018 | 1.027 | 1.88·(0.95,2.81) | 1 | Largest reduction in heterogeneity |
| Yang·2023 | 2.931 | 2.97·(1.43,4.50) | 1 | Main finding ·unchanged |
| Jin·2020 | 3.498 | 2.76·(1.09,4.43) | 1 | Main finding ·unchanged |
| Yang·2021 | 3.591 | 2.54·(0.84,4.23) | 1 | Main finding ·unchanged |
| Loubani·2021 | 2.944 | 2.96·(1.42,4.50) | 1 | Main finding ·unchanged |
| Song·2018 | 3.553 | 2.50·(0.82,4.18) | 1 | Main finding ·unchanged |
| Wang·2021 | 3.316 | 2.60·(1.11,4.08) | 1 | Main finding ·unchanged |
| Bellens·2020 | 3.052 | 2.60·(1.17,4.02) | 1 | DCT·node ·removed;DRI·unchanged |

Table S4. Fixed-effect versus random-effects network meta-analysis

Subjective cognitive function

| **Comparison** | **Random-effects SMD (95% CI)** | **Fixed-effect SMD (95% CI)** |
| --- | --- | --- |
| DCT vs CON | 0.13 (−0.22,0.47) | 0.09 (−0.12,0.31) |
| DRI vs CON | 0.44 (−0.17,1.04) | 0.41 (−0.01,0.84) |
| DPBI vs CON | 0.44 (0.03,0.85) | 0.43 (0.15,0.71) |
| DEI vs CON | 2.53 (1.58,3.47) | 2.53 (1.79,3.26) |
| DMI vs CON | 0.61 (−0.10,1.31) | 0.61 (0.22,0.99) |

Objective cognitive function

| **Comparison** | **Random-effects SMD (95% CI)** | **Fixed-effect SMD (95% CI)** |
| --- | --- | --- |
| DCT vs CON | 0.21 (−3.27,3.68) | 0.21 (−0.37,0.79) |
| DRI vs CON | 2.60 (1.17,4.02) | 1.91 (1.67,2.16) |
| DEI vs CON | 1.48 (−2.00,4.96) | 1.48 (0.87,2.10) |
| DMI vs CON | 1.56 (−0.43,3.56) | 1.30 (1.05,1.56) |
